# Comparison of diagnostic image modalities for the detection of Achilles tendon tendinopathy using ankle magnetic resonance imaging

**DOI:** 10.3389/fphys.2025.1550799

**Published:** 2025-05-23

**Authors:** Joohyun Lee, Jee Young Lee, Keum Nae Kang, Soyoon Park, Jae Ni Jang, Sukhee Park, Young Uk Kim

**Affiliations:** ^1^ Department of Anesthesia and Pain Medicine, CHA Ilsan Medical Center, CHA University, Ilsan, Republic of Korea; ^2^ Department of Korean Medicine, Integrative Cancer Center, CHA Ilsan Medical Center, CHA University, Ilsan, Republic of Korea; ^3^ Department of Anesthesiology and Pain Medicine, National Police Hospital, Seoul, Republic of Korea; ^4^ Department of Anesthesiology and Pain Medicine, Catholic Kwandong University, College of Medicine, International ST. Mary’s Hospital, Incheon, Republic of Korea

**Keywords:** Achilles tendon, cross-sectional area, thickness, diagnostic test (MeSH), magnetic resonance

## Abstract

**Background:**

A thickened Achilles tendon (AT) is one of the important morphological changes observed in Achilles tendinopathy (ATTP). Previous research studies have demonstrated that both Achilles tendon thickness (ATT) and Achilles tendon cross-sectional area (CSA) (ATCSA) are correlated with ATTP in subjects. However, the comparative value of ATT and ATCSA in relation to ATTP is not clear, and no studies have calculated the optimal clinical threshold values of ATT and ATCSA. The goal of this research was to assess ATT and ATCSA and determine which parameter is more sensitive in predicting ATTP.

**Methods:**

AT lesions were studied in 31 subjects with ATTP and 36 asymptomatic subjects who underwent ankle magnetic resonance imaging (A-MRI) and showed no evidence of ATTP. Axial T1-weighted A-MRI images were obtained at the AT level. We measured the ATT and ATCSA at the junction of the soleus and gastrocnemius aponeurosis using an image analysis program. The ATT was defined as the thickest point at the AT margin. The ATCSA was defined as the total cross-sectional area of the AT at the region showing the most pronounced inflammatory lesions. In addition, a subgroup analysis by sex was performed to evaluate the gender-specific diagnostic performance of ATT and ATCSA.

**Results:**

The average ATT was 3.83 ± 0.76 mm in the control group and 5.42 ± 0.97 mm in the ATTP group. The average ATCSA was 46.49 ± 7.12 mm^2^ in the control group and 82.59 ± 29.71 mm^2^ in the ATTP group. ATTP subjects had significantly higher ATT (p < 0.001) and ATCSA (p < 0.001) than the control subjects. ROC curve analysis showed that the optimal threshold value of the ATCSA was 57.20 mm^2^. The responsiveness of ATCSA was 87.1%, and its precision was 88.9%. The optimal threshold value of the ATT was 4.64 mm. The responsiveness of ATT was 80.6%, and its precision was 80.6%. We compared the area under the curve (AUC) for two analyzed diagnostic methods. The ATCSA’s AUC is 0.95 (95% CI: 088–1.00), and the ATT’s AUC is 0.91 (95% CI: 0.84–0.97).

**Conclusion:**

Although both ATCSA and ATT were significantly correlated with ATTP, the ATCSA was a more sensitive measurement parameter.

## 1 Introduction

The Achilles tendon (AT) is the strongest and biggest tendon in the body. The AT has the capacity to resist tensile forces. The AT stems from the soleus muscle and a distal confluence of the gastrocnemius and inserts at the bottom of the calcaneus (de Sa et al., 2018; Qahtani and Mirza, 2016). Achilles tendinopathy (ATTP) is a common overuse injury caused by excessive compression and repetitive energy storage and release (Matthews et al., 2020; Li et al., 2020). These mechanical forces can lead to sudden damage or, in the worst case, can cause AT rupture. In both cases, a stiff AT or a lack of flexibility can increase the risk of these injuries. Different methods to measure ATTP have been developed. The methods can be divided into four groups depending on modality, namely, clinical, radiographs, ultrasonography (US), and magnetic resonance imaging (MRI) (Couppe et al., 2020; Kruse et al., 2017; Syha et al., 2014). MRI facilitates the assessment of the pathologic findings of the AT and other associated pathologic conditions in the ankle joint (Sadek et al., 2015; Dallaudiere et al., 2019; Ernat et al., 2019). Most physicians consider the ankle magnetic resonance imaging (A-MRI) findings when assessing morphological changes in the AT when choosing among management options. Previous research studies evaluated the AT using a single measurement method at approximately the “halfway” point of the AT (Chulvi-Medrano et al., 2020; Genkel et al., 2020). However, an asymmetrical tear and partial thickening of the AT can occur anywhere. Therefore, a measurement mistake could occur frequently. In contrast to the Achilles tendon thickness (ATT), the Achilles tendon cross-sectional area (ATCSA) does not consider this measurement mistake because the ATCSA measures the CSA of the whole AT (Kruse et al., 2017; Intziegianni et al., 2017; Sponbeck et al., 2017). However, these parameters have not been compared. Thus, this study compared ATT and ATCSA between ATTP subjects and normal controls using A-MRI to determine which is more sensitive.

## 2 Methods and material

### 2.1 Patients

The study was retrospective and approved by the Ethical Committee of the Catholic Kwandong Institute of Incheon. We reviewed the study population who visited our orthopedic clinic from September 2014 to June 2020 and those who were diagnosed with ATTP.

The inclusion criteria were as follows: 1) a history of stiffness in the Achilles tendon; 2) ankle magnetic resonance imaging taken within 3 months of the first outpatient visit and available for review; 3) increasing pain, usually at the back of the leg or heel; 4) swelling at the back of the ankle; and 5) tenderness when touching the tendon. Subjects with any of the following disorders were excluded from the study: 1) previous ankle surgery, 2) chronic ankle instability, 3) hindfoot varus, and 4) any neuromuscular disease. All enrolled patients had clinical and imaging features consistent with mid-portion Achilles tendinopathy, with pain localized at 2–7 cm proximal to the calcaneal insertion. Patients with insertional tendinopathy were excluded.

A total of 31 individuals who met the enrollment criteria were included after a diagnosis of ATTP was confirmed by a board-certified, experienced musculoskeletal specialist.

There were 20 (64.5%) male participants and 11 (35.5%) female participants, with an average age of 40.42 ± 12.93 years (ranging from 20 to 65 years) (Table 1). All subjects underwent ankle magnetic resonance imaging. To compare the ATT and ATCSA between the study population with and without ATTP, we enrolled a control group who underwent ankle magnetic resonance imaging and showed no evidence of ATTP from September 2014 to June 2020. We enrolled subjects who did not suffer from ATTP-related symptoms in the control group. In the control group, 36 individuals (21 male participants and 15 female participants) were enrolled with an average age of 39.28 ± 15.37 years (ranging from 18 to 78 years).

**TABLE 1:** Comparison of the characteristics of the control and ATTP groups.

Variable	Control group n = 36	ATTP group n = 31	Statistical significance
Gender (male/female)	21/15	20/11	NS
Age (yrs)	39.28 ± 15.37	40.42 ± 12.93	NS
BMI (kg/m^2^)	23.2	23.8	NS
ATT (mm)	3.83 ± 0.76	5.42 ± 0.97	p < 0.001
ATCSA (mm^2^)	46.49 ± 7.12	82.59 ± 29.71	p < 0.001

Data represent the mean ± standard deviation (SD) or the numbers of patients. BMI, body mass index; ATTP, Achilles tendinopathy; ATT, Achilles tendon thickness; ATCSA, Achilles tendon cross-sectional area; NS, not statistically significant (p > 0.05).

### 2.2 Imaging parameters

A-MRI was performed using a 3.0 T MAGNETOM Skyra magnetic resonance imaging system (Siemens Medical Systems, Erlangen, Germany) and Ingenia 3.0 T (Philips, Eindhoven, Netherlands) scanners. All participants were positioned supine with the ankle secured using a strap. We acquired axial T1W fat-saturated images with an intersection gap of 0.9 mm, a slice thickness of 3 mm, a repetition time 684 ms/echo sequence time of 12 ms, a 30-cm field of view, a 448 × 291 acquisition matrix, and >3 ETL.

### 2.3 Image analysis

**FIGURE 1:**
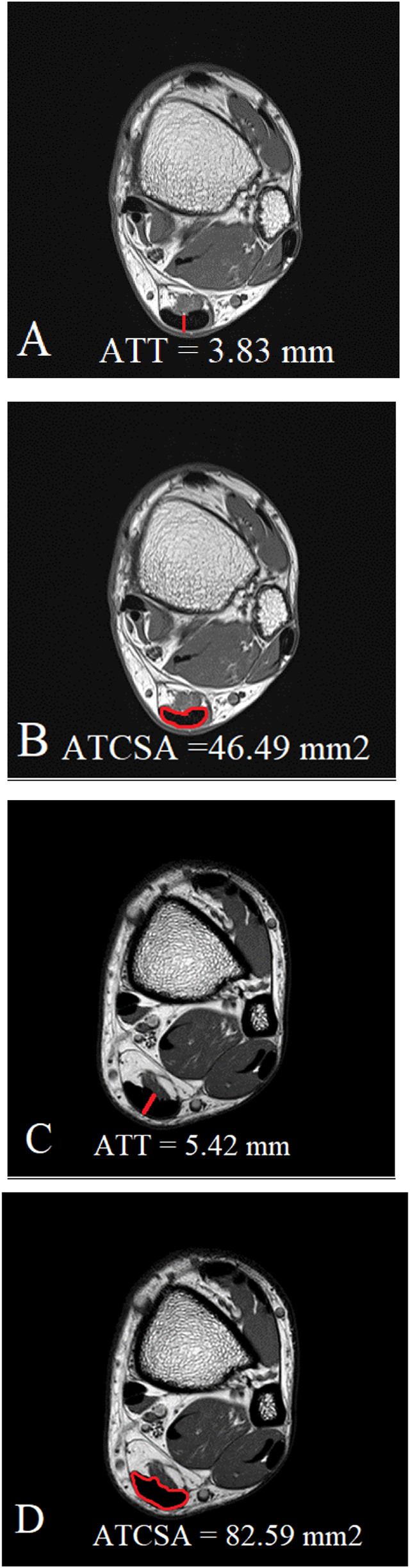
Measurement of both ATT **(A)** and ATCSA **(B)** in the normal control group was carried out on MR T1-weighted images. In Achilles tendinopathy, we measured both the thickened ATT **(C)** and thickened ATCSA **(D)** on MR T1-weighted images.

All measurements were independently performed by two experienced musculoskeletal imaging experts. We conducted both intra- and inter-rater reliability testing. The intra-class correlation coefficient (ICC) for intra-rater reliability was 0.92 for ATT and 0.95 for ATCSA. The ICC for inter-rater reliability was 0.89 for ATT and 0.93 for ATCSA, indicating excellent agreement. We acquired T1W axial MR images at the thickest point of the AT. We analyzed the ATCSA and ATT on magnetic resonance imaging using a PACS system (INFINITT; Healthcare, Incheon, South Korea) (Figure 1). We measured the ATT at the junction of the soleus and gastrocnemius aponeurosis. The ATT was measured at the AT margin. The ATCSA was measured as the cross-sectional ligament area of the AT.

### 2.4 Statistical analysis

A *post hoc* power analysis using the ATCSA difference between groups (mean ± SD: 82.59 ± 29.71 vs. 46.49 ± 7.12 mm^2^) showed that the statistical power was 0.87 at α = 0.05. Data were expressed as the standard deviation (SD) and mean value. We compared the ATCSA and ATT between the ATTP and control groups using unpaired t-tests. A ROC curve was generated to evaluate the validity of ATCSA and ATT in the diagnosis of ATTP. We used options in the SPSS package for the calculation of area under the curve (AUC), responsiveness, and precision with 95% confidence intervals (CIs) and cut-off values. p-values less than 0.05 were considered statistically significant. We used SPSS Statistics for Windows, version 22.0, for statistical data analysis (IBM Corp., Armonk, NY, United States). Normality of the data was confirmed using the Shapiro–Wilk test (p > 0.05 for all variables), justifying the use of parametric testing.

## 3 Results

**FIGURE 2:**
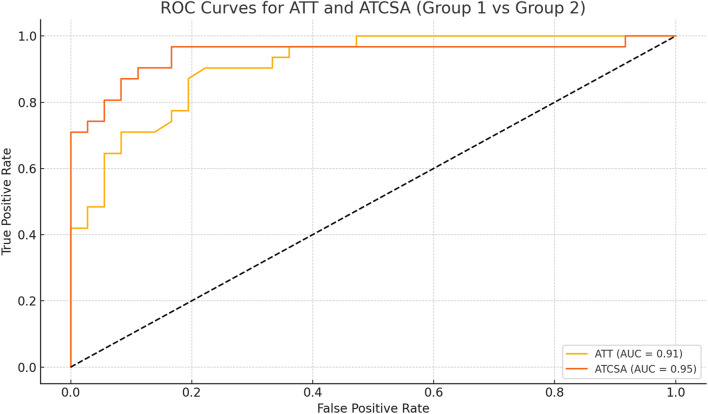
ROC curve of ATT and ATCSA for predicting ATTP. The best cut-off point of ATT was 4.64 mm and that of ATCSA was 57.20 mm^2^, with a sensitivity of 80.6% versus 87.1%, a specificity of 80.6% versus 88.9%, and an AUC of 0.91 versus 0.95, respectively. ATT, Achilles tendon thickness; ATCSA, Achilles tendon cross-sectional area. ATT AUC (95% CI) = 0.91 (0.84–0.97). ATCSA AUC (95% CI) = 0.95 (0.88–1.00). AUC, area under the curve.

**FIGURE 3:**
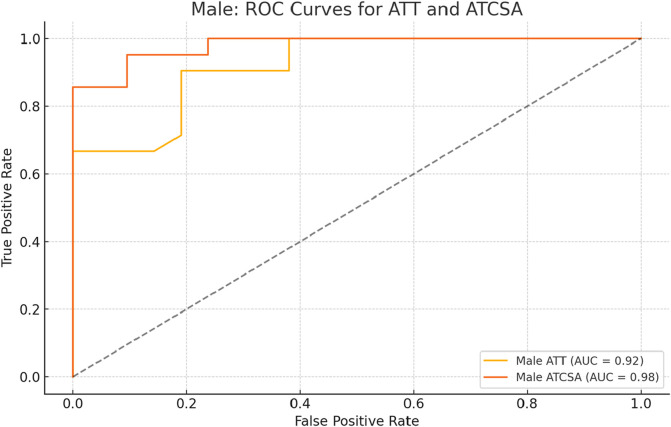
ROC curve analysis of ATT and ATCSA in male subjects. The optimal cut-off for ATT was 4.44 mm, yielding a sensitivity of 90.3% and a specificity of 77.8%. For ATCSA, the optimal cut-off was 52.13 mm^2^, with a sensitivity of 96.8% and a specificity of 83.3%. ATCSA demonstrated superior diagnostic performance compared to ATT in male participants. ATT, Achilles tendon thickness; ATCSA, Achilles tendon cross-sectional area.

**FIGURE 4:**
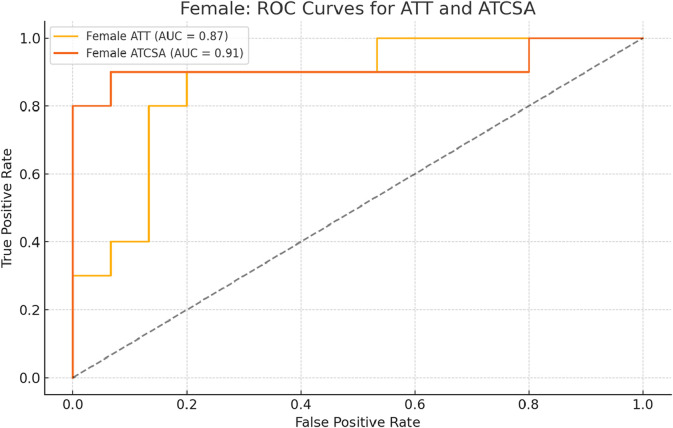
ROC curve analysis of ATT and ATCSA in female subjects. The optimal cut-off for ATT was 4.56 mm, resulting in a sensitivity of 81.8% and a specificity of 86.7%. For ATCSA, the best cut-off was 56.90 mm^2^, with a sensitivity of 90.9% and a specificity of 86.7%. ATCSA also showed better diagnostic performance than ATT in female participants. ATT, Achilles tendon thickness; ATCSA, Achilles tendon cross-sectional area.

The findings of this study demonstrated that the ATCSA, compared to the ATT, had a higher diagnostic accuracy for detecting ATTP. The observed sensitivity and specificity values indicate that ATCSA could serve as a reliable diagnostic parameter. There was no statistically significant difference in body mass index (BMI) between the two groups; the average BMI was 23.8 kg/m^2^ in the ATTP group and 23.2 kg/m^2^ in the control group. The average ATT was 3.83 ± 0.76 mm in the control group and 5.42 ± 0.97 mm in the ATTP group. The average ATCSA was 46.49 ± 7.12 mm^2^ in the control group and 82.59 ± 29.71 mm^2^ in the ATTP group. ATTP subjects had significantly higher ATT (p < 0.001) and ATCSA (p < 0.001) than the control subjects (Table 1). The ROC curve analysis showed that the optimal threshold value of the ATCSA was 57.20 mm^2^. The responsiveness of ATCSA was 87.1%, and its precision was 88.9% (Table 2). The optimal threshold value of the ATT was 4.64 mm. The responsiveness of ATT was 80.6%, and its precision was 80.6% (Table 3). We compared the AUC for the two analyzed diagnostic methods. The ATCSA’s AUC was 0.95 (95% CI: 088–1.00), and the ATT’s AUC was 0.91 (95% CI: 0.84–0.97) (Figure 2). We further performed a sex-specific subgroup analysis to assess whether the diagnostic performance of ATT and ATCSA varies by gender. For male participants, the optimal cut-off value for ATT was 4.44 mm, yielding a sensitivity of 90.3% and specificity of 77.8%. The corresponding ATCSA cut-off was 52.13 mm^2^, with a sensitivity of 96.8% and specificity of 83.3%. Among female participants, the optimal ATT threshold was 4.56 mm (sensitivity: 81.8%; specificity: 86.7%), while the best ATCSA cut-off was 56.90 mm^2^ (sensitivity: 90.9%; specificity: 86.7%). These findings suggest that although ATCSA remains a superior diagnostic marker in both sexes, the optimal thresholds may differ by gender. Two additional ROC curves illustrating male and female subgroup performance have been added to Figures 3, 4, respectively.

**TABLE 2:** Sensitivity and specificity of each cut-off point of the ATCSA.

ATCSA (mm^2^)	Sensitivity (%)	Specificity (%)
31.97	100	2.8
37.46	100	8.3
44.91	96.8	38.9
57.20^a^	87.1	88.9
60.36	74.2	94.4
69.96	67.7	100

^a^ The best cut-off point on the ROC curve; ATCSA, Achilles tendon cross-sectional area.

**TABLE 3:** Sensitivity and specificity of each cut-off point of the ATT.

ATT (mm)	Sensitivity (%)	Specificity (%)
2.41	100	2.8
3.08	100	13.9
3.81	96.8	52.8
4.64^a^	80.6	80.6
4.79	71.0	86.1
5.29	41.9	97.2

^a^ The best cut-off point on the ROC curve; ATT, Achilles tendon thickness.

## 4 Discussion

These results highlight the clinical importance of ATCSA as a superior predictor of ATTP compared to ATT. Previous studies have focused primarily on ATT; however, ATT alone may fail to capture the overall degenerative changes occurring throughout the AT. In contrast, ATCSA accounts for the entire cross-sectional area, providing a more comprehensive assessment. This is particularly valuable in cases of partial tears or asymmetric thickening, where localized measurements such as ATT may be insufficient. Moreover, the findings align with the existing literature suggesting that MRI-based measurements outperform ultrasound in detecting subtle degenerative changes.

To the best of our knowledge, the optimal cut-off value for predicting ATTP using ATT and ATCSA has not yet been evaluated. Therefore, the objective of this research was to investigate the reliability of ATT and ATCSA measurements of the AT using an A-MRI. Eventually, this study found that the ATCSA was a more suitable measurement parameter for ATTP than ATT.

ATTP means persistent tendon dysfunction and pain, which is related to mechanical loading. Although there are a lot of models to explain the pathogenesis of ATTP, the continuum model is widely used to clinically diagnose and describe ATTP (Li et al., 2020; Zhuang et al., 2019). The tendon pathology used both clinical features and imaging to characterize the three distinct stages of ATTP, namely, tendon dysrepair, reactive tendinopathy, and degenerative tendinopathy. Although the pathology is described in three different stages, it is demonstrated that ATTP pathology occurs on a continuum, with continuity between the stages (Li et al., 2020; Nazerali et al., 2013; Okochi et al., 2017). The end stage is correlated with AT rupture. Thus, early and exact diagnosis is very important.

A clinical diagnosis of ATTP is initially derived from clinical examinations and patient history. Clinical examinations have been proven to be sensitive for assessing ATTP. A-MRI and ultrasound (US) are the most frequently used imaging modalities to evaluate both the ATT and ATCSA (Dallaudiere et al., 2019; de-la-Cruz-Torres et al., 2020; Stenroth et al., 2019). Both modalities are well-known non-invasive diagnostic methods to detect ATTP. US is both sensitive and accurate for evaluating pathological structural change within AT, but it does not always correlate with AT dysfunction and pain. Although many previous reviews have demonstrated both a dissociation and association between the function and tendon structure, structural changes found through US can be considered a risk factor for the development of symptomatic ATTP. Recently, the relative risk of developing pain in asymptomatic ATTP (where clinical tests are negative but structural changes are present on US) ATTP has been frequently reported. This suggests that although the standardization of criteria for evaluating AT structural changes improves the diagnostic accuracy, there is still significant heterogeneity in US-based diagnostic techniques (Kudron et al., 2020).

A-MRI facilitates the assessment of the pathologic findings of the AT and other associated pathologic conditions in the ankle joint (Dallaudiere et al., 2019; Ernat et al., 2019). Most doctors consider the A-MRI findings when assessing morphological changes in the AT when choosing among management options. Previous research studies evaluated the AT using a single measurement method at approximately the “middle” point of the AT (Qahtani and Mirza, 2016; Pozarowszczyk et al., 2018). However, an asymmetrical tear and partial thickening of the AT can occur anywhere. Therefore, a measurement mistake could occur frequently. In contrast to the ATT, the ATCSA does not consider this measurement mistake because the ATCSA measures the CSA of the whole AT. The quantification of ATCSA is important in this study as it is a variable needed for the calculation of AT stress. However, these parameters have not been compared.

In the current research, we concluded that the ATT had 80.6% responsiveness, 80.6% precision, and an AUC of 0.91 (95% CI: 0.84–0.97) to predict ATTP. In contrast, the ATCSA had 87.1% responsiveness, 88.9% precision, and an AUC of 0.95 (95% CI: 0.88–1.00). These findings mean that the ATCSA is a better predictor of ATTP than the ATT. In addition, we used T1W A-MR images because tendons appear clearly as hypointense anatomies on T1W sequences. T1W images provide good structural details at the sites of pathology, such as in AT injuries (Filho et al., 2009).

Our current study has several limitations. First, various methods for assessing ATTP, such as US or stress radiography, have been proven to effectively discriminate cases of ATTP. In particular, US plays a very important role in the diagnosis of various diseases (Chang et al., 2024; Choi and Oh, 2024; De Cassai et al., 2024; Fouad et al., 2021; Genc et al., 2024; Heo et al., 2024; Keles et al., 2024; Kose et al., 2023a; Kose et al., 2023b; Lee et al., 2024; Li et al., 2024; Moon, 2024; Sahoo et al., 2024; Saracoglu et al., 2024; Yildiz et al., 2023). However, we only assessed the analysis of the ATT and ATCSA on A-MRI. Second, ATTP was evaluated and classified using two categories: “thin or rupture” and “thickened.” We focused only on the thickened Achilles tendon. The ligament undergoes the inflammation and proliferation phase for several months in the recovery process after the injury. Meanwhile, the ligament may show thickening due to sprain. This phenomenon has already been revealed in a study of athletics.

In addition, recent advances in artificial intelligence (AI) present promising avenues for future research. Convolutional neural networks (CNNs), in particular, hold potential for automating the extraction and classification of Achilles tendon abnormalities on MRI. Previous studies in dermatology and musculoskeletal imaging have demonstrated the effectiveness of deep learning in medical image analysis (Park et al., 2024; Iqbal et al., 2021; Iqbal et al., 2020). Integrating AI into tendon imaging may improve diagnostic accuracy and reproducibility. CNNs are capable of identifying complex morphological and textural features—such as signal heterogeneity and tendon thickening—that could complement conventional imaging biomarkers such as ATT and ATCSA.

## 5 Conclusion

In conclusion, this study establishes ATCSA as a more sensitive and precise imaging parameter for diagnosing ATTP. Clinicians should prioritize ATCSA measurements when assessing patients with suspected tendinopathy to improve diagnostic accuracy. Future research should focus on longitudinal studies to evaluate the progression of ATTP and its response to treatment using these imaging parameters. Although both ATT and ATCSA were significantly associated with ATTP, the ATCSA was a more sensitive measurement parameter than ATT. When assessing subjects with ATTP, physicians should carefully analyze the ATCSA rather than the ATT.

## Data Availability

The original contributions presented in the study are included in the article/supplementary material; further inquiries can be directed to the corresponding author.
